# A Pertussis Outer Membrane Vesicle-Based Vaccine Induces Lung-Resident Memory CD4 T Cells and Protection Against *Bordetella pertussis*, Including Pertactin Deficient Strains

**DOI:** 10.3389/fcimb.2019.00125

**Published:** 2019-04-26

**Authors:** María Eugenia Zurita, Mieszko M. Wilk, Francisco Carriquiriborde, Erika Bartel, Griselda Moreno, Alicja Misiak, Kingston H. G. Mills, Daniela Hozbor

**Affiliations:** ^1^Laboratorio VacSal, Facultad de Ciencias Exactas, Instituto de Biotecnología y Biología Molecular (IBBM), CCT-CONICET La Plata, Universidad Nacional de La Plata, La Plata, Argentina; ^2^School of Biochemistry and Immunology, Trinity Biomedical Sciences Institute, Trinity College Dublin, Dublin, Ireland; ^3^Facultad de Ciencias Exactas, Instituto de Estudios Inmunológicos y Fisiopatológicos (IIFP), CCT-CONICET La Plata, Universidad Nacional de La Plata, La Plata, Argentina

**Keywords:** *Bordetella pertussis*, pertussis, outer membrane vesicles, T_RM_ cells, pertactin deficient strains, protection

## Abstract

Pertussis is a respiratory infectious disease that has been resurged during the last decades. The change from the traditional multi-antigen whole-cell pertussis (wP) vaccines to acellular pertussis (aP) vaccines that consist of a few antigens formulated with alum, appears to be a key factor in the resurgence of pertussis in many countries. Though current aP vaccines have helped to reduce the morbidity and mortality associated with pertussis, they do not provide durable immunity or adequate protection against the disease caused by the current circulating strains of *Bordetella pertussis*, which have evolved in the face of the selection pressure induced by the vaccines. Based on the hypothesis that a new vaccine containing multiple antigens could overcome deficiencies in the current aP vaccines, we have designed and characterized a vaccine candidate based on outer membrane vesicle (OMVs). Here we show that the OMVs vaccine, but not an aP vaccine, protected mice against lung infection with a circulating pertactin (PRN)-deficient isolate. Using isogenic bacteria that in principle only differ in PRN expression, we found that deficiency in PRN appears to be largely responsible for the failure of the aP vaccine to protect against this circulating clinical isolates. Regarding the durability of induced immunity, we have already reported that the OMV vaccine is able to induce long-lasting immune responses that effectively prevent infection with *B. pertussis*. Consistent with this, here we found that CD4 T cells with a tissue-resident memory (T_RM_) cell phenotype (CD44^+^CD62L^low^CD69^+^ and/or CD103^+^) accumulated in the lungs of mice 14 days after immunization with 2 doses of the OMVs vaccine. CD4 T_RM_ cells, which have previously been shown to play a critical role sustained protective immunity against *B. pertussis*, were also detected in mice immunized with wP vaccine, but not in the animals immunized with a commercial aP vaccine. The CD4 T_RM_ cells secreted IFN-γ and IL-17 and were significantly expanded through local proliferation following respiratory challenge of mice with *B. pertussis*. Our findings that the OMVs vaccine induce respiratory CD4 T_RM_ cells may explain the ability of this vaccine to induce long-term protection and is therefore an ideal candidate for a third generation vaccine against *B. pertussis*.

## Introduction

Although vaccination is the most cost-effective strategy to prevent life threatening infectious diseases, these diseases continue to be the main cause of global morbidity and mortality (Plotkin, 2005). Vaccine preventable diseases (e.g., measles, tetanus, diphtheria, polio, etc.) are responsible for ~25% of the 10 million deaths that occur annually among children under 5 years of age. In addition, around 25% of adult deaths (15–59 years) are still attributed to infectious diseases. Therefore, there is an urgent need to improve some traditional vaccines of the Expanded Program on Immunization and to design new vaccines for emerging, re-emerging or resurgent pathogens (WHO, 2010a,b). One of the traditional vaccines that needs to be improved is that designed to prevent the severe respiratory disease whooping cough or pertussis. During the last decades the incidence of this disease has increased in adults, adolescents, as well as in children despite good vaccine coverage (Berti et al., 2014; Clark, 2014). Individuals at the extreme ends of life may be the most vulnerable to severe *Bordetella pertussis* (the etiologic agent of the disease) infection, though hospitalization can be necessary across all age groups (Mbayei et al., 2018).

The incidence of pertussis was drastically reduced following introduction of global immunization with the whole cell pertussis (wP) vaccine, consisting in a suspension of the causative agent *B. pertussis* killed by heat and chemically detoxified (Cherry, 1984). The wP vaccine was introduced in the 1940 and 1950s and it is still in use, in developing countries for the pediatric population. However safety concerns with wP vaccines (Desauziers et al., 2004; Klein, 2014) and its acceptance diminished in different countries (Romanus et al., 1987; Klein, 2014). This lead to development of acellular pertussis (aP) vaccines containing purified antigenic protein components of *B. pertussis* (2, 3, or 5 immunogens) (Sato and Sato, 1985; Edwards and Karzon, 1990). The aP vaccines have a better safety profile and gradually replaced wP vaccine in many industrialized countries (Zhang et al., 2012).

During the last two decades the epidemiology of pertussis has changed (Clark, 2014; Tan et al., 2015), with major outbreaks in many developing countries but also in developed countries (Hozbor et al., 2009; Clark, 2012), even in those with high rates of vaccination (He and Mertsola, 2008; Anon, 2010; Clark, 2014; Mbayei et al., 2018). There have been a number of explanations for the resurgence of pertussis, including waning of immunity induced by vaccines, in particular aP vaccines (Koepke et al., 2014; McGirr and Fisman, 2015), pathogen adaptation to escape vaccine induced immunity (Mäkelä P. H., 2000; King et al., 2001; Mooi et al., 2001; He et al., 2003; David et al., 2004; Gzyl et al., 2004; Bottero et al., 2007; Bowden et al., 2016), and the failure of pertussis vaccines, in particular aP vaccines, to prevent infection and spread of *B. pertussis*.

Regarding pathogen evolution, the first reports were related to polymorphism in genes coding for proteins included in the vaccine [pertactin (PRN) and pertussis toxin (PTx) among others] and later in the pertussis toxin promoter (*ptx*P) (Advani et al., 2011; Kallonen et al., 2012). Recently, there has been an increase in *B. pertussis* isolates that do not produce some of the vaccine antigens (Bodilis and Guiso, 2013; Hegerle and Guiso, 2014; Lam et al., 2014). In particular in US, Canada and Australia it was reported that PRN-deficient isolates [PRN(-)] increased substantially in the last years (Lam et al., 2014; Pawloski et al., 2014; Tsang et al., 2014). These isolates are expected to be resistant to the phagocytosis mediated by anti-pertactin antibodies (Hellwig et al., 2003). It has been proposed that the loss of this vaccine antigen probably provides a selective advantage for bacterial survival in populations vaccinated with aP vaccines. Commercial aP vaccines containing PTx, PRN, and filamentous hemagglutinin (FHA) are not as effective as expected in controlling the infection caused by the recent circulating bacteria that do not express PRN (Hegerle et al., 2014). Moreover, recently it was demonstrated in a mixed infection mouse model that PRN(-) *B. pertussis* colonizes the respiratory tract of aP immunized mice more effectively than the PRN(+) strain, out-competing the PRN(+) strain (Safarchi et al., 2015).

Regarding waning immunity, it is well known that while wP vaccines induce potent Th1 and Th17 responses, the current aP vaccines are inefficient at promoting Th1 responses, but do induce potent antibody and Th2-polarized responses and weak Th17 responses (Ross et al., 2013; Brummelman et al., 2015). Furthermore, immunization with wP vaccines appear to be more effective than current aP vaccines at inducing immunological memory and in conferring long-term protection against pertussis (Brummelman et al., 2015). Recent data has demonstrated that wP but not aP vaccines induced CD4 T memory cells that reside in the lungs (Allen et al., 2018; Borkner et al., 2018). These respiratory tissue-resident memory CD4 T cells that express CD44^+^CD62L^low^CD69^+^ confer long-term protective immunity against *B. pertussis*.

Possible solutions to improved control of pertussis disease, in the short term, include the return to the use of the wP vaccine in the primary dose and/or add more boosters. However, adding many boosters implies having both, resources to sustain the vaccination schedule and pharmaceuticals capable of responding to the demands of all countries that require them. Regarding those countries that switched to aP, it seems difficult to reintroduce the same wP vaccine since the acceptability of this vaccine by the population had been lost (Plotkin, 2014).

We have designed a new multi-antigen aP vaccine formulation that shares the beneficial properties of current aP vaccines in terms of biosafety and those of wP vaccines in terms of immunogenicity and protective capacity (International patent granted in the USA and in process in other countries, Application Number: PCT/IB2014/060143) (Roberts et al., 2008; Asensio et al., 2011; Gaillard et al., 2014). This new acellular formulation has been obtained from membrane components of *B. pertussis* (outer membrane vesicles, OMVs) in which antigens are presented in their native conformation, with membrane-associated PAMPs acting as immunostimulatory molecules, such as in the commercial wP vaccines. We have reported that the OMVs-based vaccine was capable of inducing a more robust immune response than current aP vaccines with a Th1/Th17 and Th2 cellular profile (Bottero et al., 2016), that confers long lasting protection against *B. pertussis* (Gaillard et al., 2014).

In this study we have evaluated whether our OMVs vaccine is capable of overcoming the deficiencies of commercial vaccines in both controlling infections caused by PRN(-) isolate/strain and inducing memory immunity. We found that our OMVs-based formulation has a higher protective capacity against the PRN(-) bacteria than that induced with a commercial aP vaccine. We found that CD4 T cells with a tissue-resident memory (T_RM_) cell phenotype (CD44^+^CD62L^low^CD69^+^ and/or CD103^+^) accumulated in the lungs of mice after the second OMVs vaccine immunization. CD4 T_RM_ cells were also detected in mice immunized with wP vaccine, but not in the animals immunized with a commercial aP vaccine. The CD4 T_RM_ cell population was significantly expanded through local proliferation following respiratory challenge of mice with *B. pertussis*. These CD4 T_RM_ cells secreted IFN-γ and IL-17 that have previously been shown to play a critical role in adaptive immunity against *B. pertussis* infection. Our findings suggest that the OMVs-vaccine is an ideal candidate for the development of a third generation pertussis vaccine.

## Materials and Methods

### Animals

C57BL/6 (8-week-old) mice were obtained from Harlan Laboratories U.K. or the Comparative Medicine Unit, Trinity College Dublin and housed in a specific pathogen–free facility. All animal experiments performed in Dublin were conducted in accordance with the recommendations and guidelines and under licenses approved by the Health Products Regulatory Authority of Ireland in accordance with protocols approved by the Trinity College Dublin Animal Research Ethics Committee. Animal experiments using female BALB/c mice with 3–4 weeks of age, obtained from the Instituto Biológico Argentino (Biol. SAIC, Argentina) were also performed in Argentina. The studies have been approved by Ethical Committee for Animal Experiments of the Faculty of Science at La Plata National University (Argentina, approval number 004-06-15 and 003-06-15).

### Bacterial Strains and Growth Conditions

*Bordetella pertussis* Tohama phase I strain, its isogenic mutant strain defective in PRN (Roberts et al., 1991), and PRN-deficient clinical isolate (Bp935) were used throughout this study. Bp935, which was kindly provided by CDC (Atlanta) is a representative PRN(-) isolate obtained from the 2012 Washington (US) outbreak (Pawloski et al., 2014). PRN deficiency in this isolate was caused by IS481 insertion into *prn* gene, and its MLVA-MLST type is the most prevalent among the isolates from that outbreak (*ptx*P3, *ptx*A1, *prn*2). Bacteria were grown on Bordet-Gengou agar (BGA, Difco) supplemented with 10% defibrinated sheep blood at 36.5°C for 72 h and plated again on the same medium for 24 h before each infection.

### Isolation and Characterization of Outer Membrane Vesicles (OMVs)

OMVs were produced and characterized as previously described (Hozbor et al., 1999; Asensio et al., 2011). Briefly, culture samples from the decelerating growth phase were centrifuged and the bacterial pellet obtained was resuspended in 20 mM Tris–HCl, 2 mM EDTA pH 8.5. The suspension was sonicated (ultrasonic bath) in cool water for 20 min. After two centrifugations at 10,000 × g for 20 min at 4°C, the supernatant was pelleted at 100,000 × g for 2 h at 4°C. This pellet was re-suspended in Tris buffer (20 mM pH 7.6). The samples obtained were negatively stained for electron microscope examination. Protein content was estimated by the Bradford method using bovine serum albumin as standard. The presence of the main immunogenic proteins in the OMVs was corroborated by immunoblot assays using specific antibodies as we previously described (Roberts et al., 2008).

### Formulation of OMV-Based Vaccine

The characterized OMVs that range in size from ~50 to 200 nanometers in diameter were used to formulate the vaccine with tetanus (5–7 Lf/ dose with a power ≥2 UIA/ml serum) and diphtheria (1–3 Lf / dose with an output of 0.1 UIA/ml serum) toxoids as we previously described (Gaillard et al., 2014). The LPS concentration determined by GC-MS ranged from 0.275 to 0.352 μg per dose of OMV based vaccine. The safety of this vaccine was evaluated by a mouse weight-gain test (WHO, 2007) and the murine and human whole-blood IL-6–release assays (Stoddard et al., 2010; Bottero et al., 2016). To perform the experiments described below we verified that the OMV based vaccine prepared by us fulfilled the WHO criteria for safety in the weight-gain test. The safety of OMV-based vaccines was also confirmed by human whole-blood assays.

### Immunization of Mice

Groups of 4–5 female C57BL/6 or BALB/c mice (discriminated in the legends to the figures) were immunized with OMVs-based vaccine formulated as previously described (3 μg total protein per dose formulated with alum as adjuvant) (Asensio et al., 2011), 1:50 or 1:10 human dose of aP vaccine BOOSTRIX® [GlaxoSmithKline, with composition per human dose: pertussis toxoid (8 μg), pertactin (2.5 μg), FHA (8 μg), tetanus toxoid (20 IU), diphtheria toxoid (2 IU), and alum as adjuvant], or 1:40 human dose of wP vaccine (National Institute of Biological Standards and Control, South Mimms, UK; NIBSC batch 41S) in 200 μl PBS via intraperitoneal (i.p.) injection using a two-dose schedule. The i.p route, although not translatable to humans, was chosen as it allowed us to compare our results with previous studies on the immunogenicity and protective OMVs-based pertussis vaccines that used this route of immunization (Gaillard et al., 2014; Bottero et al., 2016). Two weeks after the second immunization, mice were sacrificed, and the immune response was evaluated in lungs and spleen. For protection assays mice were challenged with *B. pertussis* by exposure to an aerosol of 5 × 10^8^ bacteria per ml or by intranasal inoculation (sublethal dose 10^7^-10^8^ CFU 40 μl^−1^) as is described below. In both case, mice were killed 1 and/or 2 weeks after challenge.

### *B. pertussis* Respiratory Challenge

For protection assays, mice were challenged with *B. pertussis* by exposure to an aerosol of 5 × 10^8^ bacteria per ml or by intranasal inoculation (sublethal dose 10^7^-10^8^ CFU 40 μl^−1^). For aerosol challenge, *B. pertussis* bacteria were grown from a frozen stock on Bordet Gengou plates containing glycerol and horse blood (Cruinn) at 36.5°C. After 3 d of culture, the bacteria were collected in supplemented Stainer–Scholte medium and cultured overnight at 36.5°C in a shaking incubator at 220 rpm. Bacteria were centrifuged and resuspended in 1% casein solution, and the OD was measured at 600 nm. *B. pertussis* infection of C57BL/6 mice was performed by aerosol challenge (BP338 strain; 1 × 108 CFU/ml) administered using a nebulizer (PARI TurboBOY SX) over 10 min (Zhang et al., 2012). The course of infection was followed by performing CFU counts on lung homogenates at intervals post-infection (p.i.), as described (Zhang et al., 2012). For intranasal challenge, mice were infected with a sublethal dose (10^7^-10^8^ CFU 40 μl^−1^) of *B. pertussis* clinical isolate/strain. Bacterial counts were performed 7 days after the challenge as described previously (Asensio et al., 2011; Gaillard et al., 2014).

### Intravascular Staining for Discriminating Circulating Cells From Lung-Retained Cells

To discriminate blood-borne circulating cells from lung-localized cells, we used a well-described approach in which anti-mouse PE-CD45 Ab (eBioscience) was administered i.v. to mice 10 min before they were euthanized and lungs were harvested (Gzyl et al., 2004).

### Isolation and FACS Analysis of Cells From Lung or Spleen Tissue

Lung and nasal tissue mononuclear cell suspensions were prepared by mechanical (chopping with a scalpel) followed by enzymatic disruption of tissue for 1 h at 37°C with Collagenase D (1 mg/ml; Sigma-Aldrich) and DNAse I (20 U/ml; Sigma-Aldrich). Next, lungs or spleens were passed through a 40-mm cell strainer to a obtain single-cell suspension, followed by RBC lysis. The cells were incubated with CD16/CD32 FcgRIII (1:100) to block IgG Fc receptors. Cells were incubated with LIVE/DEAD Aqua (Invitrogen), followed by surface staining with fluorochrome-conjugated anti-mouse Abs for various markers. To detect cytokines, cells were stimulated with PMA (50 ng/ml) and ionomycin (500 ng/ml) in the presence of brefeldin A (5 mg/ml) for 4 h at 37°C. The following surface Abs were used: CD45R-PE, CD3-BV421, CD44-BV605 (Biolegend), CD62L-PE-CF594 (BD), CD103-BV786, CD4-APC-eF780, CD69-FITC (eBioscience). For detection of intracellular cytokines, cells were fixed in 2% PFA and permeabilized with 0.5% saponin (Sigma-Aldrich, Ireland), followed by staining with IL-17A–PerCP-Cy5.5 and IFN-γ-BV650 (eBiosciences). Fluorescence minus one or non-specific isotype Abs were used as controls. Flow cytometric analysis was performed on an LSR Fortessa, and data were acquired using Diva software (BD Biosciences). The results were analyzed using FlowJo software (TreeStar).

### Ag-Specific IL-17, IL-5, and IFN-γ Production by Spleen Cells

Spleens from untreated and immunized mice were passed through a 40-mm cell strainer to obtain a single-cell suspension. Spleen cells were cultured with sonicated *B. pertussis* (sBp; 5 μ/ml), or medium only. After 72 h of incubation, IFN-γ, IL-5, and IL-17A concentrations were quantified in supernatants by ELISA. sBp was obtained by sonication of the bacterial suspension in PBS (10^10^ CFU/ml) on ice using ultrasonic homogenizer (10 × 15 s. pulse; Sonopuls Bandelin). The sonicated bacterial suspension was then centrifuged at 10,000 rpm × 15 min. Supernatant was collected and protein concentration was determined as described above.

### Statistical Analysis

For the analysis of CFU counts in animal lungs, before applying the statistical methods described below we evaluated the normality of the data by using Shapiro-Wilk test (http://scistatcalc.blogspot.com.ar/2013/10/shapiro-wilk-test-calculator.html). After verifying that our CFUs data follows a normal distribution, we statistically analyzed them by using one-way analysis of variance (ANOVA) followed by Bonferroni's multiple comparison test (GraphPadPrism®). Differences were considered to be significant when *p* < 0.05. For fluorescence values analysis, we used Mann-Whitney statistical analysis (*p* < 0.05). All statistical analysis of data was performed using GraphPadPrism® version 6.00 for Windows, GraphPad® Software.

## Results

### OMVs Vaccine Protects Against PRN(-) *B. pertussis* Clinical Isolate

**Figure 1:**
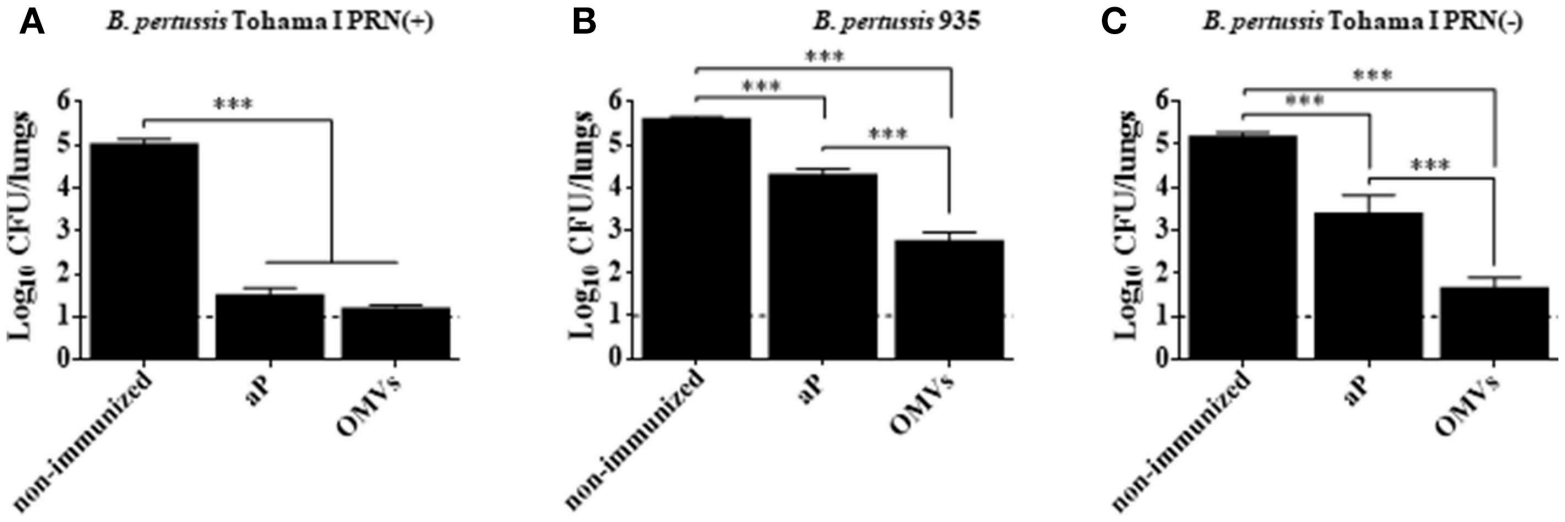
Protection against *B. pertussis* PRN(-) isolate induced with an OMVs vaccine in a mouse model. BALB/c mice were immunized (i.p.) twice, 2 weeks apart. Mice were challenged with sublethal doses (5 × 10^7^ CFUs/40 μl) of *B. pertussis* Tohama I PRN(+) **(A)**, *B. pertussis* 935 PRN(-) **(B)** or *B. pertussis* Tohama I PRN(-) **(C)** 2 weeks after the second immunization with aP or OMVs vaccine. Three independent experiments were performed for each strain/isolate. Results from one representative experiment are shown. Results depicted are means of 5 mice per group at 7 days post-challenge. The dashed line indicates the lower limit of detection. The number of bacteria recovered from mouse lungs is expressed as the log 10 means ± SEM (error bars) of colony forming units (CFU) per lung. Data obtained were analyzed statistically by using one-way analysis of variance (ANOVA) followed by Bonferroni's multiple comparison test (GraphPadPrism®). In **(A,B,C)** ^***^Significant differences with *p* < 0.001.

We compared the protective capacity of the OMVs vaccine with that induced with a commercially available aP vaccine against infection with *B. pertussis* Tohama phase I strain [*ptx*P1 variant and *prn*1 allele, PRN(+)] or PRN(-) clinical isolate of *B. pertussis* Bp935. The OMVs vaccine and the commercial aP vaccine conferred protective immunity against the *B. pertussis* Tohama phase I strain. The bacterial counts in the lungs were significantly lower in immunized compared with non-immunized mice (Figure 1A). In contrast, the protective capacity of OMVs vaccine against PRN(-) isolate was significantly higher than that of the commercial aP vaccine (Figure 1B). Mice immunized with the OMVs had 3 Log_10_ reduction in colonies 7 days after challenge with *B. pertussis* PRN(-) compared with the non-immunized mice, whereas the CFU counts in the lungs of mice immunized with the commercial aP vaccine was only reduced by 1 log_10_ (Figure 1B). Since there may be other unidentified features other than the absence of PRN in the clinical isolate, we also evaluated the efficacy of the studied acellular vaccines against *B. pertussis* Tohama phase I isogenic bacteria that only differ in PRN expression. We found that immunization with the OMVs vaccine significantly (*p* < 0.001) reduced the bacterial counts in the lung to near the detection limit of the assay after challenge with the PRN-defective *B. pertussis* Tohama mutant (Figure 1C). Importantly, the protective capacity of commercial aP vaccine was impaired (at least in 1 log_10_, *p* < 0.001) against PRN defective mutant strain (Figure 1C) compared with that observed again the wild type strain (Figure 1A). The findings demonstrate that the current aP vaccine has impaired capacity to protect against mutant strain or certain circulating strains of *B. pertussis* that lack PRN.

### CD4 T_RM_ Cell Accumulate in the Lungs of Mice Immunized With the OMVs Vaccine

**Figure 2:**
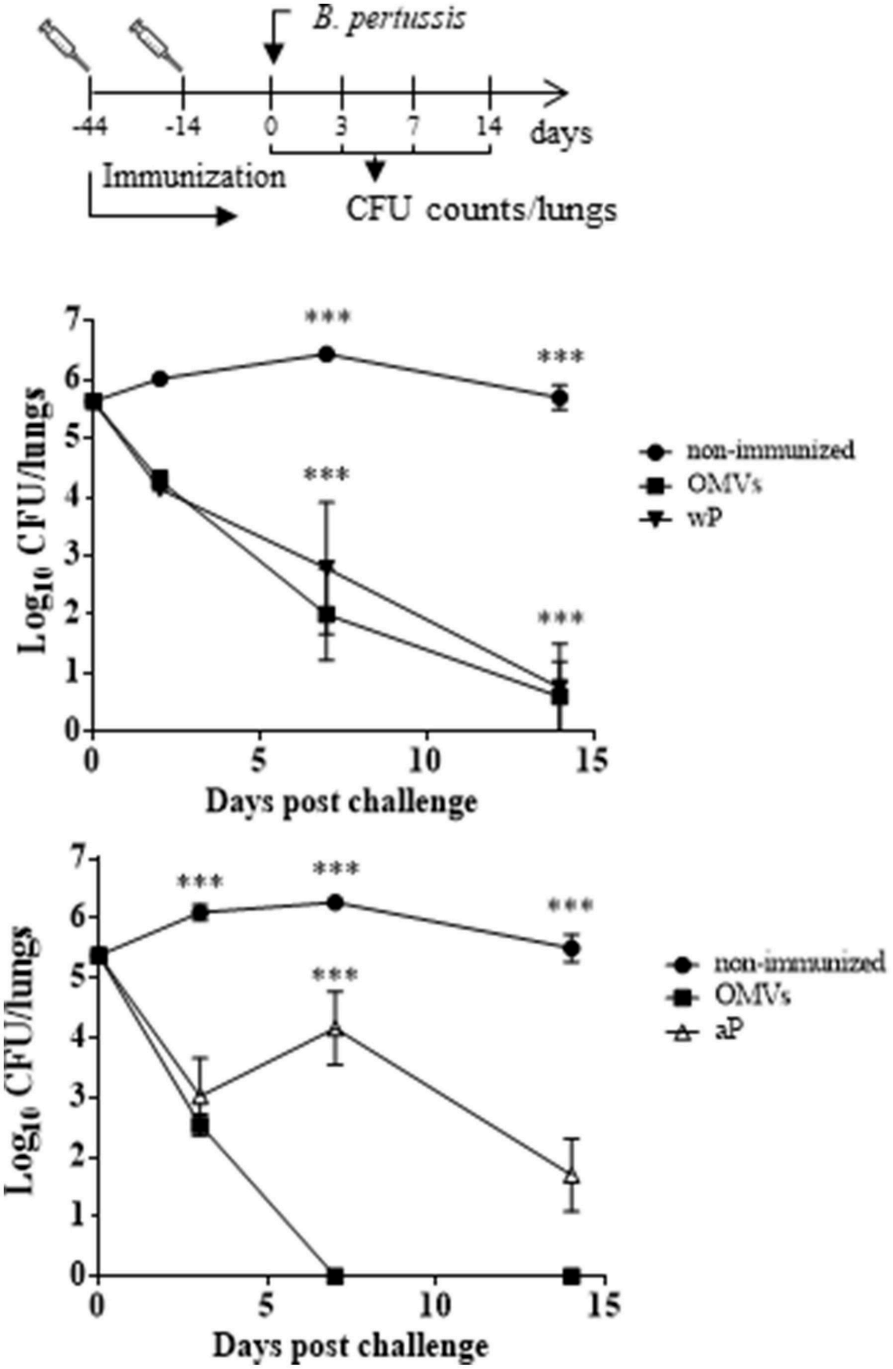
Protection of mice against *B. pertussis* infection induced with the OMVs vaccine compared with wP and aP vaccines. C57BL/6 mice were immunized (i.p.) twice, 4 weeks apart, with either wP, aP, or OMVs vaccines or were non-immunized. Mice were challenge 2 weeks after the second immunization by aerosol with *B. pertussis* BP338 strain (1 × 10^8^ CFU/ml) administered using a nebulizer (PARI TurboBOY SX) over 10 min. Results are shown as the number of bacteria recovered from the mouse lungs at different time points, expressed as the log_10_ of the means ± SEM (error bars) of the CFUs per lung, plotted on the *ordinate* for the samples from the lungs of the experimental groups indicated on the *abscissa*. The experiment involved three biological replicates with the results from a representative one being presented. The dotted horizontal line marks the lower limit of detection. ^***^p < 0.001 (ANOVA followed by the Bonferroni *post-hoc* test).

We used the murine model to evaluate whether the OMVs-based candidate pertussis vaccine induced systemic antigen-specific immune responses and CD4 T cells with a T_RM_ cell phenotype in lungs. The Mills group had already demonstrated that natural infection or immunization with an wP vaccine primed respiratory T_RM_ cells, whereas an aP vaccine did not (Wilk et al., 2017; Allen et al., 2018). Therefore, we performed experiments comparing the responses with that induced with the OMV-based vaccine with aP or wP vaccines, with the wP vaccines acting as positive control. First, we used the murine respiratory challenge model to confirm that the immunization with the different vaccines tested in this study were capable of inducing protection against infection (Figure 2). We found that two parenteral immunizations with the OMVs vaccine was highly effective at preventing lung infection; the CFU counts were at or close to background 7 and 14 days post-challenge (Figure 2). The protection induced with the OMVs vaccine was similar to that generated with an wP vaccine (Figure 2 upper panel) and was significantly better than induced with the aP vaccine (Figure 2, lower panel).

**Figure 3:**
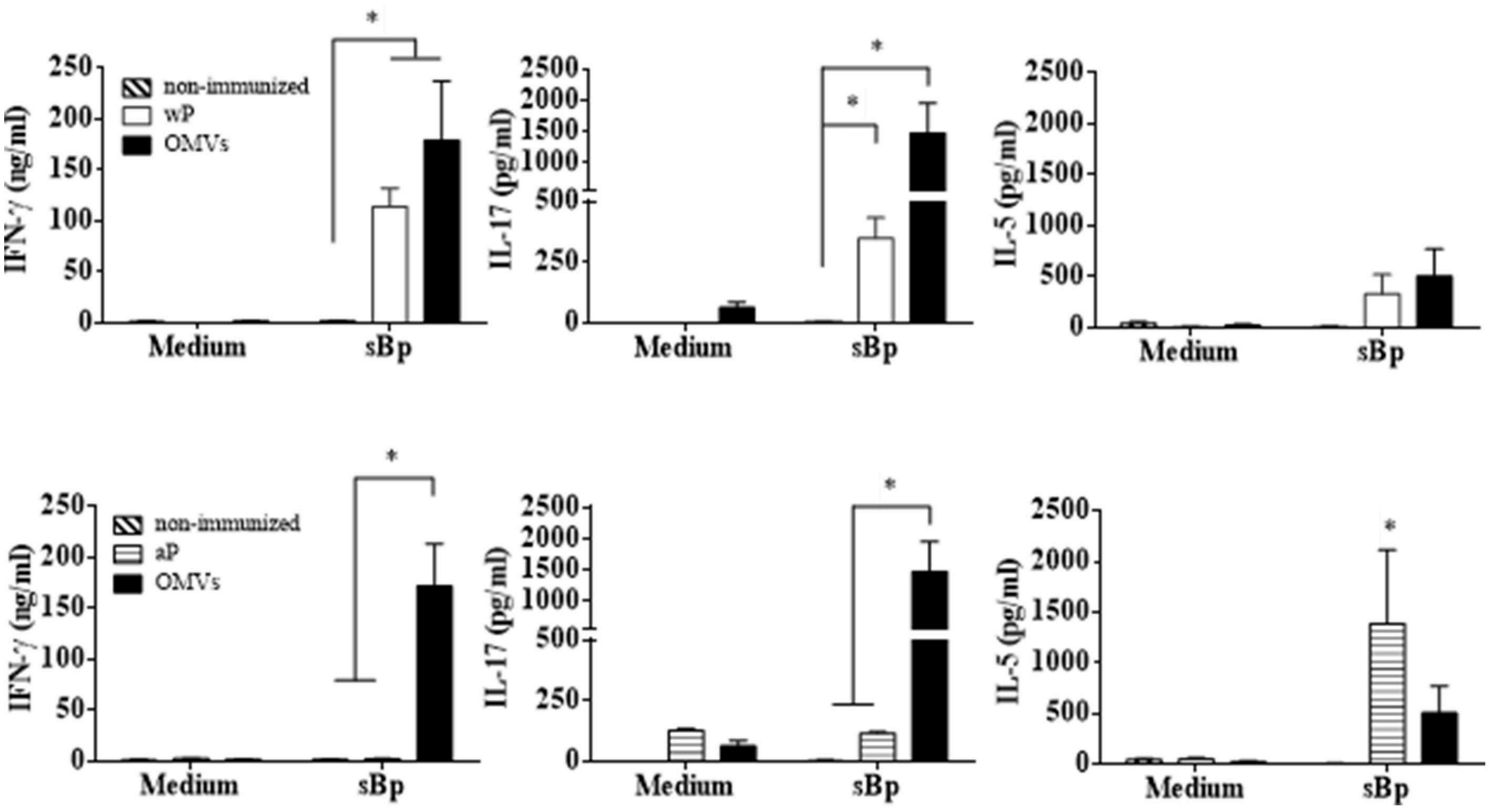
Cytokine production by spleen cells from immunized mice. C57BL/6 mice were immunized as described in Figure 2. Fourteen days after the last immunization, mice were sacrificed and their spleen cells stimulated with sonicated *B. pertussis* (sBp) or medium alone (negative control). After 72 h of culture, the concentrations of IFN-γ, IL-17, and IL-5 were determined in the culture supernatant by ELISA. The results are expressed as mean values (± SEM) of three biological replicates with each having 4 mice per group. Significant differences were analyzed for each cytokine between non-immunized and immunized mice. ^*^p < 0.05, ANOVA followed by the Bonferroni *post-hoc* test.

To examine antigen-specific immune responses to *B. pertussis*, spleen cells from immunized mice 14 days after the second dose were stimulated with sonicated *B. pertussis* (sBp), cytokines were quantified in the supernatants by ELISA. The results revealed that spleen cells from mice immunized with the OMVs vaccine produced significantly higher concentrations of IFN-γ and IL-17 than spleen cells from mice immunized with the aP vaccine (Figure 3). *Bordetella pertussis*-specific IFN-γ and IL-17 was also detected in spleen cells from mice immunized with the wP vaccines. However, antigen-specific IL-17 production was significantly stronger in mice immunized with the OMVs vaccine. In contrast, the aP vaccine predominantly induced antigen-specific IL-5. These data demonstrate that OMVs and wP vaccine induce a mixed Th1/Th17 response while the current aP vaccines induce Th2-polarized responses.

**Figure 4:**
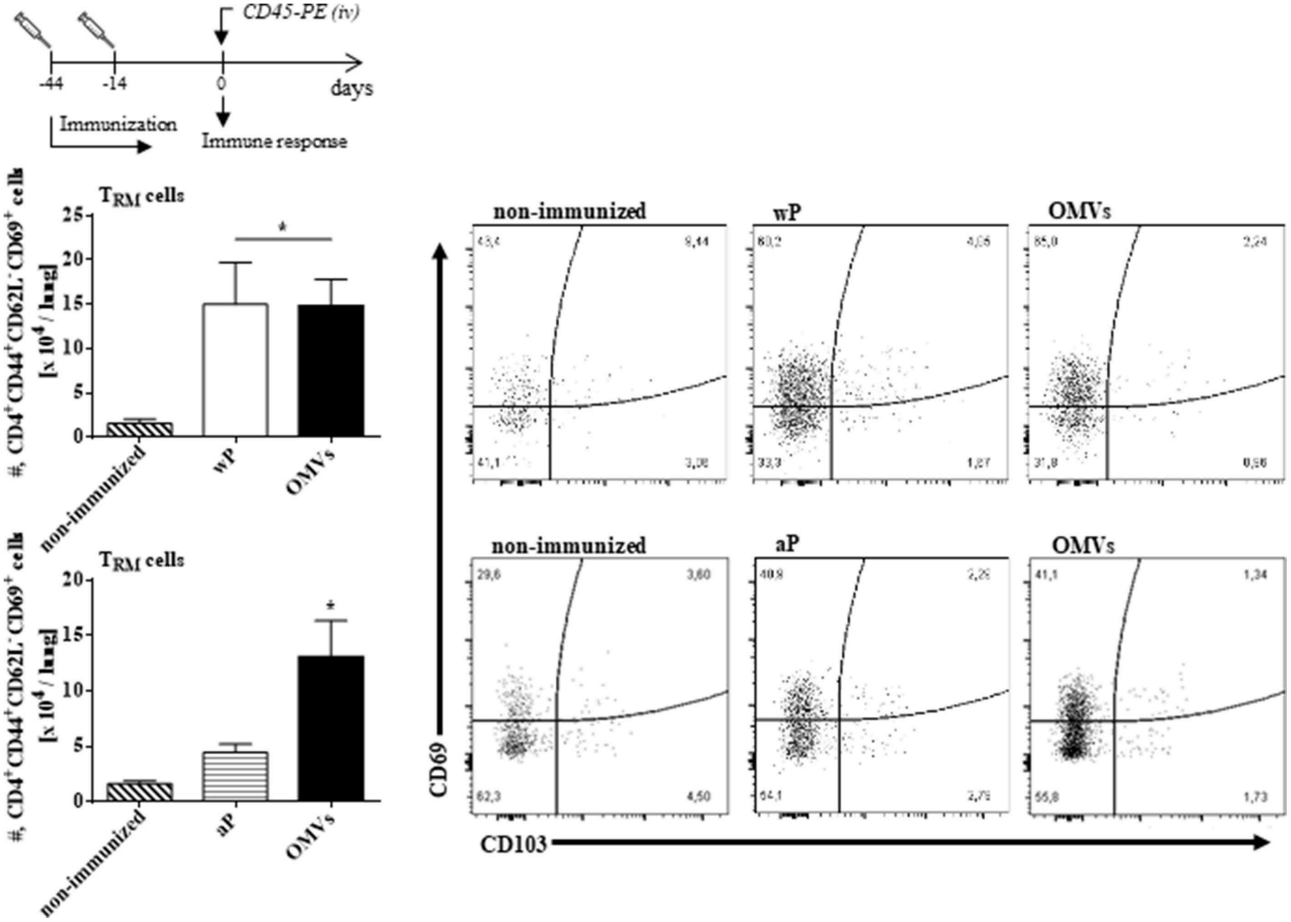
OMVs and wP vaccines induce CD4 T_RM_ cells in lungs. C57BL/6 mice were immunized as described in Figure 2. Fourteen days post-immunization, mice were i.v. injected with anti-CD45-PE antibody 10 min before euthanasia. Lung tissue was taken from all mice and the T cell response was analyzed by flow cytometry. Only tissue-resident cells (CD45-PE negative) were included in the analysis. Absolute counts of CD4^+^ T_RM_ (CD45–, CD44+, CD62L^−^, CD69+, CD103+/–, CD4+). Data are mean ± SEM (*n* = 4 mice) for CD4^+^ T_RM_ cells from lungs. ^*^p < 0.05, two-way ANOVA with Bonferroni multiple-comparison test. Representative flow cytometry plots are shown on the right.

**Figure 5:**
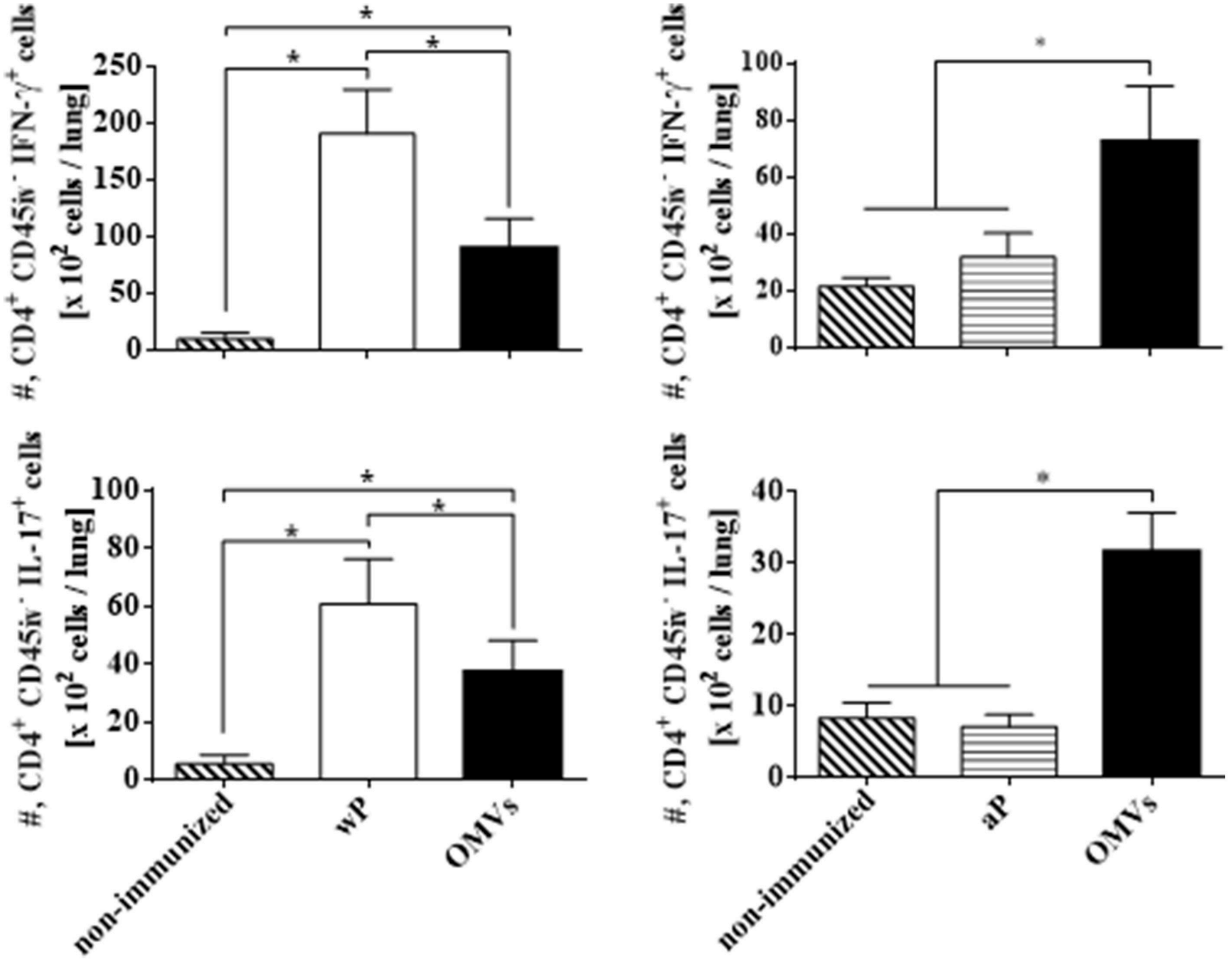
IFN-γ and IL-17 production by CD4 T_RM_ cells in lungs of immunized mice on the day of challenge. C57BL/6 mice were immunized as described in Figure 2 and CD4 T_RM_ were quantified in the lungs 2 weeks after the 2nd immunization (the day before challenge) by i.v. labeling with anti-CD45 as described in Figure 3. Intracellular cytokine staining was performed to quantify IFN-γ and IL-17-secreting T_RM_ cells. Only tissue-resident cells (CD45-PE negative) were included in the analysis. Results are absolute number of IFN-γ-producing CD4^+^ CD45^−^ T cells and IL-17-producing CD4^+^ CD45^−^ T cells in the lung. The bars indicate the mean ± SEM. ^*^p < 0.05, two-way ANOVA with Bonferroni multiple-comparison test.

**Figure 6:**
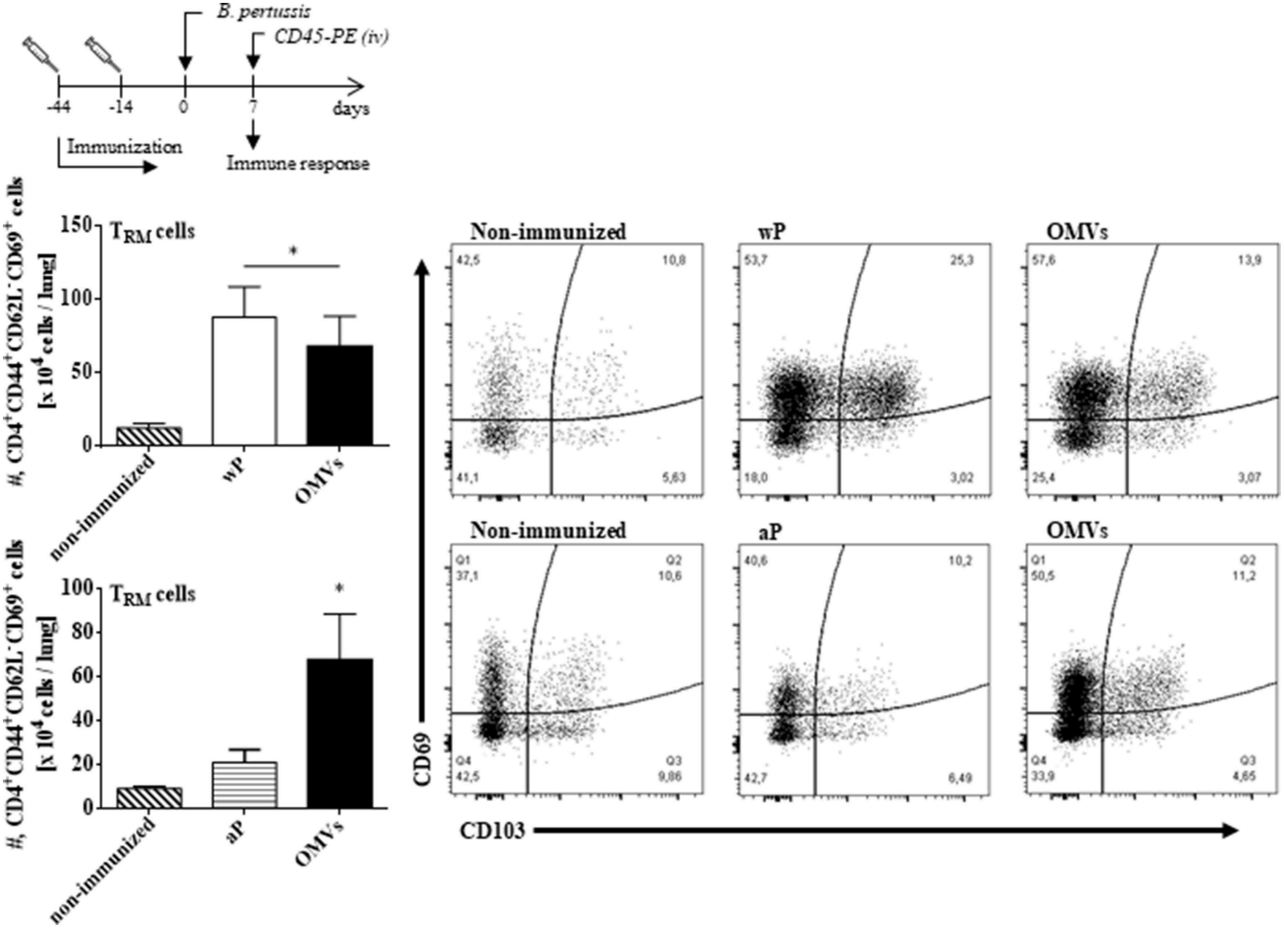
CD4 T_RM_ cells in lungs of immunized mice after challenge with *B. pertussis*. C57BL/6 mice were immunized as described in Figure 2 and CD4 T_RM_ were quantified in the lungs of immunized mice 7 days post-*B. pertussis* challenge as described in Figure 4. Absolute counts of CD4^+^ T_RM_ (CD45–, CD44+, CD62L^−^, CD69+, CD103+/–, CD4+) are represented. Data are mean ± SEM (*n* = 4 mice) for CD4^+^ T_RM_ cells from lungs. ^*^p < 0.05, two-way ANOVA with Bonferroni multiple-comparison test. Representative flow cytometry plots are shown on the right.

We next assessed T_RM_ cell in the lungs (the gating strategy is shown in Figure S1). In order to discriminate blood borne circulating leukocytes from lung-retained leukocytes, we used a validated approach (Gzyl et al., 2004) in which a fluorescently labeled anti-CD45 antibody (Ab) was administered i.v. to mice 10 min prior to sacrifice. Circulating cells become labeled with the Ab (CD45^+^), whereas the Ab cannot penetrate the tissue to stain the lung resident cells and therefore remain unstained (CD45^−^). We found that the OMVs vaccine and the wP vaccine (used as a positive control) induced cytokine-secreting respiratory CD4 T_RM_ cells. There were significant higher number of lung-resident CD4 T cells that expressed the T_RM_ cell markers CD69 and/or CD103 between in the lungs of mice immunized with OMVs compared with aP immunized or non-immunized mice (14.02 × 10^4^ ± 2.09 × 10^4^ T_RM_ for OMVs, 14.99 × 10^4^ ± 4.70 × 10^4^ T_RM_ for wP, 4.44 × 10^4^ ± 0.76 × 10^4^ T_RM_ for aP, 1.59 × 10^4^ ± 0.26 × 10^4^ T_RM_ for PBS; Figure 4). To evaluate the functionality of the induced T_RM_ cell we used intracellular cytokine staining for IFN-**γ** or IL-17. The results showed that the immunization with the wP vaccine or the OMVs vaccine induced IFN-γ or IL-17-secreting CD4 T_RM_ cells in the lungs. Although the numbers were highest in mice immunized with the wP vaccine, the number of cytokine-secreting CD4 T_RM_ cells significantly (*p* < 0.05) higher in the lungs of mice immunized with the OMVs vaccine when compared with the aP vaccine (Figure 5). Furthermore, CD4 T cells with a tissue-resident memory phenotype expanded significantly (5-fold) after *B. pertussis* challenge (Figure 6) in mice vaccinated with the wP or OMVs-based vaccine, but not in mice immunized with the aP vaccine (Figure 6). These findings demonstrate that immunization of mice with the OMVs vaccine, like the wP vaccine but not the aP vaccine, is capable of generating T_RM_ cells that expand in the lung after *B. pertussis* challenge.

## Discussion

In this study we demonstrated that the OMVs vaccine, like the wP vaccine, promoted induction of protective immunity against *B. pertussis* lung infection in mice and that while both vaccines were effective at inducing *B. pertussis*-specific INF-γ (marker of Th1 cells) and IL-17 (marker of Th17 cells) by spleen cells, the aP vaccine mainly induced IL-5 (marker of Th2 cells) (Ryan et al., 1998; Bottero et al., 2016). Though antibodies are known to be involved in protection against pertussis, Th1 and Th17 responses are also crucial for mediating adaptive immunity and clearance of *B. pertussis* from the respiratory tract (Mills et al., 1993; Ryan et al., 1997).

Consistent with previous studies showing that OMV-based vaccine was capable of conferring both long-lasting immunity and protection against pertussis (Gaillard et al., 2014), here we demonstrated that OMV-based vaccine was also effective at inducing respiratory INF-γ- and IL-17-secreting T_RM_ cells. T_RM_ cells were also expanded in the lungs of mice immunized with the wP vaccine, not in mice immunized with a commercial aP vaccine. The failure of aP vaccine to induce T_RM_ cells, even using the i.p. route of vaccination, which induces the strongest immune response in mice, may explain the waning immunity reported in populations immunized with this vaccine (Klein et al., 2013; McGirr and Fisman, 2015). The growing evidences that pertussis affects all age groups suggests that pertussis vaccines, particularly aP vaccines, do not provide long-lasting immunity. The induction of respiratory T_RM_ cells in immunized mice with the OMVs vaccine is an important finding since this memory CD4 T cell population not only provide much more immediate protection than the anti-pathogen responses conferred by naïve T-cells, they also could provide more effective immune protection to the host as demonstrated in other models of infection (Teijaro et al., 2011; Glennie et al., 2017). CD4 T_RM_ cells have been shown to play a crucial role in protective immunity against re-infection with *B. pertussis* (Wilk et al., 2017) and following vaccination with aP vaccine formulated with a novel adjuvant, comprising agonists for TLR2 and intracellular receptor stimulator of interferon genes (STING) (Allen et al., 2018). Our experiments with the OMV focused on lung T_RM_ cells, but we do not rule out the possibility that OMV induces T_RM_ cells in upper respiratory tract or in the gut. Thus, the immunization with the OMVs as well as wP vaccine induces long-term memory T cells that could spread to different parts of the body and a fraction of these cells would constitute the respiratory TRM cell population. We found that the CD69^+^CD4^+^ T_RM_ cells induced in the lungs by immunization with OMVs or wP vaccines secreted INF-γ and IL-17. Moreover, these CD69^+^CD4^+^ T_RM_ cells were expanded in the lungs after *B. pertussis* challenge in mice immunized with OMVs or wP vaccine, but not in mice immunized with a commercial aP vaccine.

The long-lived IL-17-producing Th17 memory T cells have also been detected in the respiratory mucosa of *B. pertussis*-infected baboons (Warfel and Merkel, 2013). The studies in this non-human primate model showed that Th17 cells persist long after pertussis infection and suggest that these cells play an important role in adaptive immunity to *B. pertussis* (Warfel and Merkel, 2013). Our findings suggest that, in contrast to the aP vaccine, immunization with the OMVs vaccine is an effective approach for inducing lung INF-γ and IL-17-secreting T_RM_ cells (present study) and for conferring long lasting protection against *B. pertussis* colonization (Gaillard et al., 2014). Although the used i.p. route of immunization is not translatable to humans, several previous studies, including our own had used this route as useful and highly reproducible route of systemic immunization as proof-of-principle in mice. However, we have reported that intranasal immunization with an aP vaccine formulated with a novel adjuvant is much more effective than the i.p. route for the generation of *B. pertussis*-specific T_RM_ cells (Allen et al., 2018). Furthermore, studies with *B. pertussis* OMV have demonstrated that pulmonary immunization is more effective than the s.c. route of immunization for induction of CD103^+^ T_RM_ cells (Raeven et al., 2018). Therefore, a “prime and pull” strategy of systemic priming followed by nasal boosting might be an interesting approach for induction of systemic and local memory immune response against *B. pertussis* (Shin and Iwasaki, 2012).

The OMVs derived from *B. pertussis* represent an attractive aP vaccine candidate (Roberts et al., 2008; Asensio et al., 2011; Ormazabal et al., 2014; Hozbor, 2016) not only because of its safety and ability to induce protective Th1, Th17 cells (Mills et al., 1993; Ryan et al., 1997) and T_RM_ cells, but because it contains a greater number of immunogens in conformations close to those found in pathogen, when compared with the current aP vaccines (Hozbor, 2016). This broad immunogenic composition is an important characteristic of our vaccine candidate since is expected to exert a lower selection pressure on the circulating bacterial population than that exerted by the commercial aP vaccines consisting of only a few antigens. The prevalence of bacteria that do not express vaccine antigens in regions that only use aP vaccine provides indirect evidence of selection pressure being exerting by the aP on the circulating bacterial population (Bodilis and Guiso, 2013; Hegerle and Guiso, 2014; Lam et al., 2014). In particular in United States, Canada and Australia it was reported that PRN(-) strains have increased substantially in recent years (Lam et al., 2014; Pawloski et al., 2014; Tsang et al., 2014). The polymorphism in PRN described first and the spread of PRN-deficient isolates later, have elicited deep concerns in the healthcare systems since it was hypothesized that these changes might represent a selective advantage of the bacteria against immunity induced by the aP vaccines. In particular PRN-deficient clinical isolates may have an advantage in an aP-vaccine primed immunity (Martin et al., 2015). It has been reported that PRN-deficient clinical isolates are able to overcome the anti-PRN mediated inhibition of macrophage cytotoxicity *in vitro*. Moreover, a recent study revealed that recent PRN-deficient *B. pertussis* clinical isolates harboring *ptx*P3 variant and *prn*2 allele remain at higher CFUs/lung and are capable of sustaining infection longer than isolates still producing this adhesin, in mice immunized with a 3-component aP vaccine mice (Hegerle et al., 2014). The authors of such study speculated that these particular isolates might thus be capable of infecting immunized individuals at an earlier stage of waning immunity post-aP vaccine immunization or post-infection, presenting an advantage when compared to isolates producing PRN. These findings (Hegerle et al., 2014) are consistent with those on the higher fitness of PRN negative strain in the immunized mice recently reported by Safarchi et al. (2015).

Consistent with previous reports (Hegerle et al., 2014; Safarchi et al., 2015), we found that immunization with commercial aP vaccine does not protect against PRN(-) isolate as effectively as against *B. pertussis* Tohama strain (PRN+). However, the PRN(-) strain used in this study was a clinical isolate that is not isogenic to *B. pertussis* Tohama strain (PRN+) and contains polymorphisms at other loci that may affect the fitness of these bacteria. Therefore, we also examined protection against a PRN defective mutant derived from *B. pertussis* Tohama strain. Consistent with previous results (Roberts et al., 1991), we showed that in addition to PRN, other virulence factors and key antigens are equally expressed in parental and derived strain. We found that the commercial aP vaccine exhibits lower level of protection against the PRN(-) strain when compared with the parental PRN(+) positive strain. These results clearly showed the impact of the absence of PRN expression in the effectiveness of aP vaccine against *B. pertussis* when comparisons are made on strains that contain the same genetic background.

The results obtained here clearly showed that the OMVs vaccine is more effective than a current commercial aP vaccine against PRN(-) strains. Therefore the OMV formulation appears as an attractive vaccine candidate that could replace the current aP without causing concern on the reactogenicity associated with wP vaccines because of the proven safety of the OMVs vaccines (Bottero et al., 2016). Since major limitations of the current aP are their strong selection pressure exerted on the circulating bacterial population and their failure to induce sustained protective immunity, the OMV-based vaccine, that contains high number of antigens and that induces INF-γ and IL17-secreting T_RM_ cells, has the potential to replace the current aP vaccine.

## Ethics Statement

This study was conducted in accordance with the recommendations and guidelines and under licenses approved by the Health Products Regulatory Authority of Ireland and Argentina. The protocol was approved by the Trinity College Dublin Animal Research Ethics Committee and the Ethical Committee for Animal Experiments of the Faculty of Science at La Plata National University (Argentina, approval number 004-06-15 and 003-06-15).

## Author Contributions

DH and KM planned the study, interpreted data, and wrote the manuscript. MZ and MW planned the study, interpreted data, and edited the figures and manuscript. FC, EB, GM, and AM performed experiments and laboratory analyses. All authors approved the final manuscript.

### Conflict of Interest Statement

The authors declare that the research was conducted in the absence of any commercial or financial relationships that could be construed as a potential conflict of interest.
