# Supplementary figures and images for: A Pertussis Outer Membrane Vesicle-Based Vaccine Induces Lung-Resident Memory CD4 T Cells and Protection Against Bordetella pertussis, Including Pertactin Deficient Strains
Source: Front Cell Infect Microbiol. 2019 Apr 26;9:125. doi: 10.3389/fcimb.2019.00125 (PMC6498398; doi:10.3389/fcimb.2019.00125)

Figure S1. Gating strategy

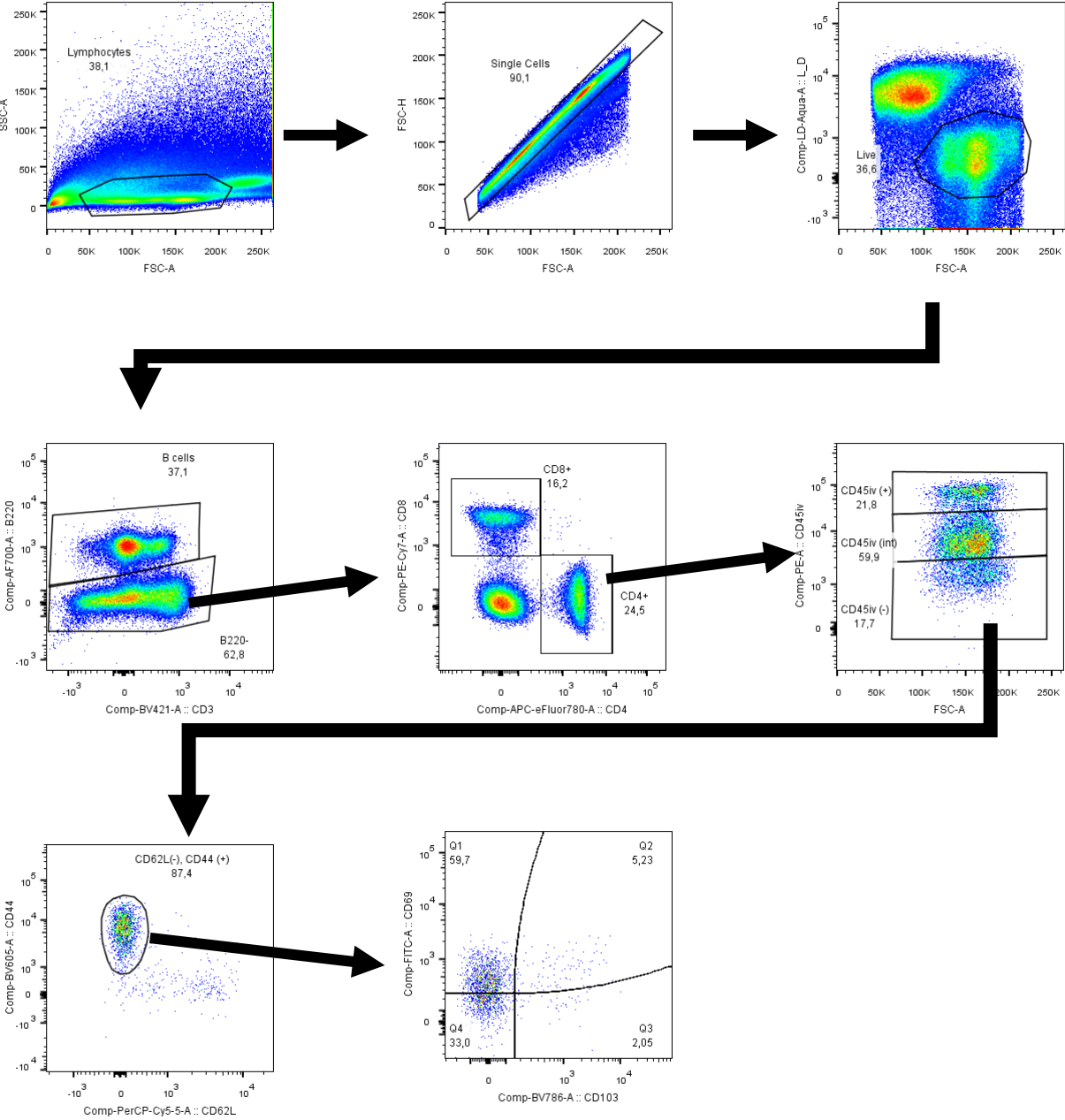

Supplement: Supplementary file 1 [file Data_Sheet_1.PDF]
